# Supplementary material for: Butyrate Protects against SARS-CoV-2-Induced Tissue Damage in Golden Hamsters
Source: Int J Mol Sci. 2023 Sep 16;24(18):14191. doi: 10.3390/ijms241814191 (PMC10532055; doi:10.3390/ijms241814191)
Supplement: Supplementary file 1 [file ijms-24-14191-s001.zip › ijms-2580951-supplementary.pdf]

Table S1: Primer sequence used for RT-qPCR.

Gene	Forward primer (5'->3')	Reverse primer (5'->3')
Ifnb1	TACTGGCAGCTGGGAAGGTA	TGCCTGCAACCATTATCCAGT
Ifnar1	TCAGCAAGTGTCGCAAGCTA	TGTGGCTGCAAGTTCTCGAT
Ifnar2	AATTCGGGGTTGTCGGCTTT	AGGTGACGTTCCCAGTGATG
Il6	AGACAAAGCCAGAGTCATT	TCGGTATGCTAAGGCACAG
Il1b	GAGAGTGTGGACCCCAAACA	TAAATCCTGGCCGCTGTTGT
Tnfa	TGAGCCATCGTGCCAATG	AGCCCGTCTGCTGGTATCAC
Icam1	CCGTGAGCTCCCATGGAAAT	TGAGGCTGAGGAGGTCTGAT
Vcam1	CCTTTCCCTCTGAGAGCGTC	TATGCGCCGTCAATGGACTT
Sele	AAGCTATGACACACCCTGCC	ATTCTGAGCTCCAACTCGCC
Nos3	CACCTCACCGTAGCTGTGTT	GTCCCTGGACCCACTAGGAT
Nos2	GACCATGGAGCATCCCAAGT	AAATTCAAGGCCACCCACCT
Ifng	TGTTGCTCTGCCTCACTCAGG	AAGACGAGGTCCCCTCCATTC
Nox2	TTGATGGACCCTTTGGCACA	AACCACTCGAAGGCATGTGT
Casp8	AATGCCGGAAGTGTGTGACT	CGTTCTTCCTCGCCTTGCTA
Casp3	AAGATCCCTGAACTCCATGTCC	CTGTGCTGGATGTTCTCCAAGT
Casp6	AGATGCCGATTGCTTCCTGT	TTCCAACCAGGCTCTGACAC
Casp7	GACCAGAGTGAACGACAGGG	ATGGTCAACGGCCGAAGTAG
Bax	TTGCTACAGGGTTTCATCCAGG	TCTCCGATTCGCCTGAGACA
Bcl2	AAATCGCCGAGAAGAAGCGA	GTTCCACGGTTTGGCTTCAC
Muc2	CAGACAATGGTGGCTGGCTA	TTGTGGATGCAGGGACACTC
Actg	ACAGAGAGAAGATGACGCAGATAATG	GCCTGAATGGCCACGTACA
